# Influence of Hyaluronic Acid Lip Filler Augmentation on Smile Design Parameters in Young Female Population: An Observational Study

**DOI:** 10.1155/ijod/2797836

**Published:** 2026-02-12

**Authors:** Ameer Biadsee, Ameen Biadsee, Haya Milhem, Matityahou Ormianer, Zeev Ormianer

**Affiliations:** ^1^ Department of Oral Rehabilitation, The Maurice and Gabriela Goldschleger School of Dental Medicine, Tel Aviv University, Tel Aviv, Israel, tau.ac.il; ^2^ Department of Otolaryngology – Head and Neck Surgery, Meir Medical Center, Sackler Faculty of Medicine, Tel-Aviv University, Tel-Aviv, Israel, tau.ac.il; ^3^ Department of Otolaryngology/Head and Neck Surgery, Hadassah Hebrew -University Medical Centre, Jerusalem, Israel, hadassah.org.il

**Keywords:** 3D facial scanning, hyaluronic acid, lip augmentation, tooth exposure

## Abstract

**Objective:**

To evaluate the effect of hyaluronic acid (HA) lip augmentation on anterior tooth exposure and smile‐line characteristics using three‐dimensional (3D) facial scanning technology.

**Materials and Methods:**

Twenty‐five female participants were enrolled. All injections were performed by a single experienced clinician following a standardized protocol, with a total of 1.0 mL of HA filler administered to the lips. 3D facial scans were obtained before treatment, immediately after injection, and at a 4‐week follow‐up. Maxillary left central incisor and canine exposure during smiling, as well as upper lip volume, were measured at each timepoint using dedicated software.

**Results:**

The mean participant age was 26.2 years, and the mean follow‐up period was 28.8 days. At follow‐up, maxillary central incisor and canine exposure decreased by 0.1 mm compared to pre‐treatment values (*p*  < 0.001). Upper lip volume increased by 0.79 mm^3^ at follow‐up (*p*  < 0.001). An inverse correlation was observed between lip volume and central incisor exposure, indicating reduced tooth display with increased lip volume. No significant correlation was found between lip volume and canine exposure or between central incisor and canine exposure.

**Conclusion:**

HA lip augmentation was associated with small but statistically significant changes in anterior tooth exposure during smiling. These findings highlight the influence of perioral soft tissue on smile‐related dental parameters and underscore the importance of considering lip volume when planning esthetic dental and restorative treatments.

## 1. Introduction

The smile is a central component of facial expression and plays a key role in conveying friendliness and positive social intent [1]. An attractive smile is closely linked to self‐esteem and interpersonal confidence and often shapes first impressions in both social and professional settings [2, 3].

The appearance of a smile is influenced by multiple anatomical and dental factors, including the smile line and arc, upper lip curvature, labiodental relationship, buccal corridors, incisal edge position, and the extent of tooth and gingival display within the orofacial window [4–6]. Together, the upper and lower lips frame this display, making them critical contributors to smile esthetics. Lip volume and proportion, in particular, play an important role in facial balance and have been shown to significantly influence perceived attractiveness [7].

In recent years, interest in minimally invasive esthetic procedures aimed at enhancing lip prominence has increased substantially [8]. Hyaluronic acid (HA) fillers are now among the most commonly performed cosmetic treatments worldwide, accounting for more than 4 million procedures in 2022 [9]. They are widely used to restore or augment lip volume due to their favorable biocompatibility and hygroscopic properties [10]. Although HA is rapidly degraded in its natural form, chemical modification allows it to remain stable for longer periods after injection.

Despite the growing popularity of lip augmentation, its influence on dental esthetics, particularly tooth display during smiling, remains insufficiently explored. One recent study using two‐dimensional photography reported no significant change in upper incisor exposure after HA augmentation; however, the method used did not allow volumetric assessment of the lips [11]. Given the increasing integration of advanced facial scanning systems in dental practice, three‐dimensional (3D) stereo photogrammetry now provides a more precise and reproducible method for evaluating soft‐tissue changes, including lip morphology [12, 13].

The aim of this study was to assess the effect of HA lip augmentation on anterior tooth exposure and smile‐line characteristics using 3D facial scanning. The null hypothesis was that HA augmentation would not produce measurable changes in tooth exposure or smile line.

## 2. Materials and Methods

The study was approved by Tel‐Aviv university Ethics Committe (Number 0006087‐5).

Eligible patients for the study were women over the age of 18 who were planning to undergo non‐surgical lip augmentation with HA fillers. Exclusion criteria included a history of any systemic disease that could interfere with filler treatment, pregnancy or breastfeeding, use of anticoagulant medications, known allergies to HA or lidocaine, and a history of lip fillers within the previous year. Twenty‐five women who met these criteria were enrolled. Each participant received a full explanation of the procedure, expected results, possible risks, and available alternatives, and informed consent was obtained. Before the injections, all makeup was removed from the face and lips.

All injections were performed by the same experienced clinician, following a standardized infiltration protocol to ensure consistency. A total of 1.0 mL of HA filler (STYLAGE Special Lips; VIVACY) was used for each patient, with 0.5 mL injected into the upper lip and 0.5 mL into the lower lip. The filler contains 18.5 mg of HA gel, 1 g of mannitol, and 0.30% lidocaine and is supplied in a 1 mL syringe with a 30‐gauge needle. The injection technique consisted of a combination of linear threading along the vermillion border and small depot injections within the body of the lip to support volume, enhance definition, and maintain natural symmetry. Care was taken to place the material in a consistent plane across all patients, avoiding overcorrection.

Three facial scans were captured for each participant using a 3D scanner (MetiSmile; Shining 3D, China) while smiling. The first scan (T0) was taken before the injection, the second (T1) immediately after the procedure, and the third (T2) at the 4‐week follow‐up visit. All scans were performed under identical conditions, including lighting, patient positioning, and the distance from the scanner.

Measurements of the exposure of the maxillary left central incisor and left canine during smiling were taken at each timepoint using the scanner’s designated software. Tooth exposure was assessed by measuring the vertical position of the upper lip relative to the incisal edges, as illustrated in Figure 1a–c. Upper lip volume was assessed using the software’s volumetric analysis feature at all three timepoints, as shown in Figure 2a–c.

Figure 1Closed area selection of the lip line cropped from the original 3‐dimensional image measuring the amount of left maxillary incisor and canine exposure while smiling at the 3 timepoints, (a) before, (b) immediately after, and (c) 4‐week follow‐up.

**Figure:**
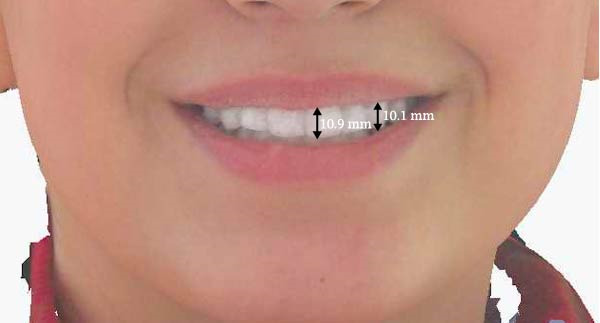
(a)

**Figure:**
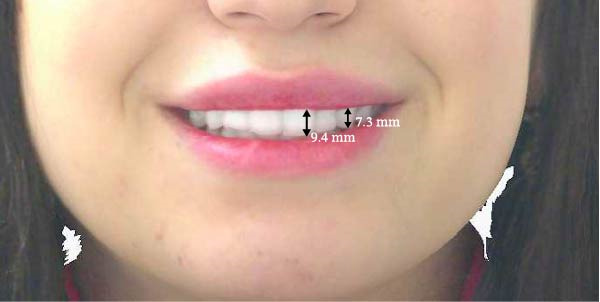
(b)

**Figure:**
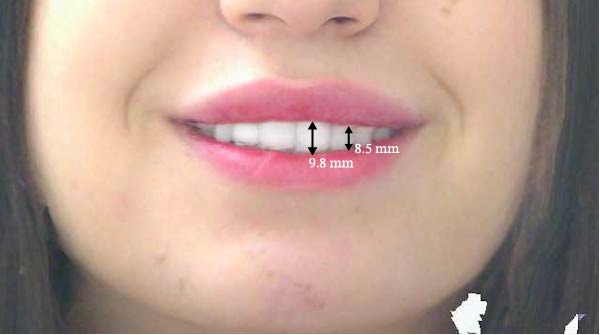
(c)

Figure 2Closed area selection of the lip line cropped from the original 3‐dimensional image measuring the upper lip volume while smiling at the 3 timepoints, (a) before, (b) immediately after, and (c) 4‐week follow‐up.

**Figure:**
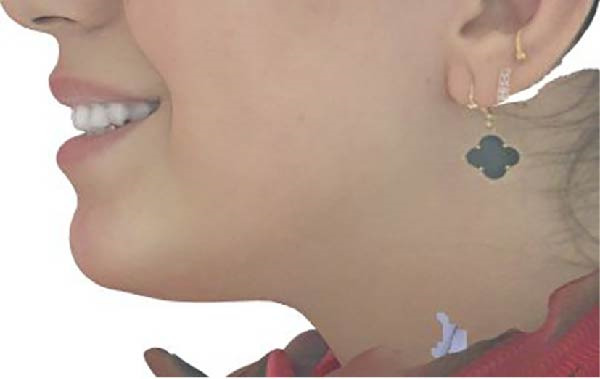
(a)

**Figure:**
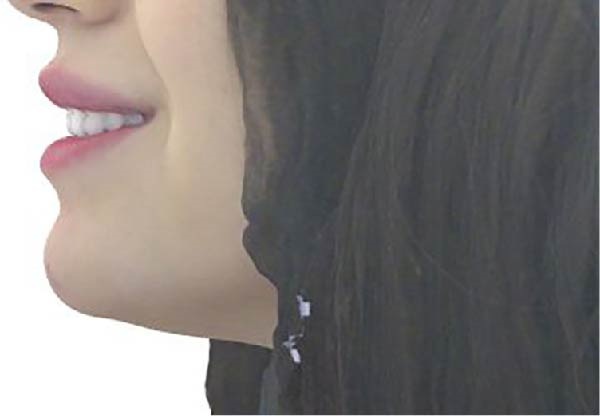
(b)

**Figure:**
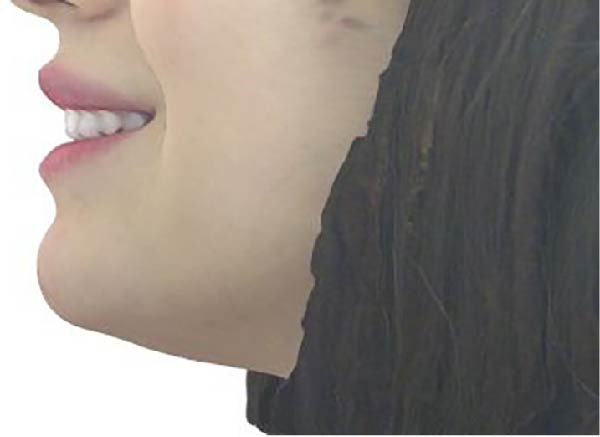
(c)

A power analysis was conducted prior to recruitment to determine an appropriate sample size. Based on expected small‐to‐moderate changes in soft‐tissue morphology and using a significance level of *α* = 0.05 with a power of 0.80, the minimum required sample was calculated to be 21 subjects. To account for possible dropouts, 25 patients were ultimately included.

Data analysis was performed using IBM SPSS Statistics for Windows, version 27. A repeated‐measures ANOVA was used to assess the effect of time on the recorded measurements. When differences were identified, Bonferroni‐adjusted pairwise comparisons were used to evaluate changes between the three timepoints. Statistical significance was set at *p*  < 0.05.

## 3. Results

Twenty‐five female patients were included in the study. The mean age of the participants was 26.2 years (range 21–33), and the mean follow‐up period was 28.8 days (range 28–32). Teeth exposure and upper lip volume measurements are summarized in Table 1.

**Table 1:** Maxillary central, canine exposure (in mm), and upper lip volume (mm) at each time point.

Location measured^a^	Pre‐treatment (T0)	Immediately after (T1)	4 weeks (T2)	*p*‐Value
Left maxillary central	8.5 ± 1.7	7.4 ± 1.7	8.4 ± 1.7	<0.001
Left maxillary canine	7.9 ± 1.6	7.2 ± 1.7	7.8 ± 1.5	<0.001
Upper lip volume	6.1 ± 1.7	7.4 ± 1.6	6.9 ± 1.6	<0.001

^a^Values are presented as mean ± standard deviation.

There were statistically significant differences between the pre‐treatment (T0), immediate post‐treatment (T1), and follow‐up (T2) measurements. Maxillary central incisor exposure decreased by 0.1 mm at follow‐up compared to pre‐treatment, 95% CI [0.041–0.213], *p* = 0.003 and by 0.1 mm for the canine, 95% CI [0.034–0.212], *p* = 0.005.

Upper lip volume increased by 0.79 mm^3^ at follow‐up compared to pre‐treatment, 95% CI [0.480–1.105], *p*  < 0.001.

The data also showed an inverse correlation between lip volume and central incisor exposure (*r* = −0.35, *p* = 0.04), suggesting that participants with greater lip volume had slightly less exposure of the central incisors during smiling. No significant correlation was observed between lip volume and canine exposure, nor between central incisor and canine exposure (*p*  > 0.05).

Repeated‐measures analysis showed that the immediate post‐injection increase in lip volume was higher than at follow‐up, likely reflecting transient swelling. This pattern suggests that while the filler increases lip volume, a small portion of the initial effect is temporary and decreases slightly over the first month. These findings provide quantitative evidence of the soft tissue changes that occur after HA lip augmentation and their subtle effects on smile parameters.

## 4. Discussion

The null hypothesis of the study, that there would be no difference in tooth exposure and smile line after HA lip augmentation, was rejected because significant differences were observed. Dental esthetics have emerged as one of the most compelling areas of interest within the field of dentistry. Esthetic dentistry focuses on creating a harmonious integration of the teeth, gums, lips, and overall facial appearance. In recent years, there has been a notable increase in the demand for minimally invasive procedures, including the administration of botulinum toxin type A and dermal fillers [8, 12].

This study provided quantitative outcomes regarding teeth exposure and lip volume after lip augmentation. One study found that HA filler increased the lip volume resulting in a more appealing smile; however, it did not provide quantitative results [13].

The findings indicate a significant reduction in central incisor and canine exposure immediately post‐procedure compared to pre‐treatment, with partial recovery at follow‐up. The results of the current study differed from another report that found that HA fillers do not alter the exposure of the upper incisors. 11 A possible explanation is the methodology. The current study used 3D facial scanning, which was found to be a valid method for measuring facial changes [10].

In this study, a total of 1 mL of HA was injected (0.5 mL per lip), which is higher than the volumes used in some previous studies that often ranged between 0.5–0.8 mL/patient. This higher volume may have contributed to the measurable changes in lip volume and the observed reduction in tooth exposure. The immediate decrease in tooth exposure could also be attributed to soft tissue swelling and the anesthetic effect of lidocaine in the syringe. Partial recovery at follow‐up indicates a lasting but moderate tissue alteration compared to pretreatment, affecting teeth exposure. A negative correlation between lip volume and central incisor exposure and no correlation to canine exposure was found, indicating that individuals with greater lip volume exhibited less central incisor exposure. This can be explained by the injection technique, which gives more attention to the Cupid’s bow. In addition, during smiling, the zygomaticus major muscle elevates the angle of the mouth, which increases canine exposure.

The utilization of a standardized injection protocol, a 1 mL syringe of HA, and the inclusion of patients with no history of filler treatments contributed to enhanced precision and reliability of the results. This relationship provides insight into the esthetic balance between soft and hard tissue dynamics, reinforcing the role of lip structure in determining smile esthetics. Clinically, this suggests that interventions affecting lip volume may inadvertently influence the tooth display and should be carefully considered in treatment planning. Future research should explore long‐term effects and include a broader population sample to validate these results. Additionally, further studies with different HA concentrations and quantities are required to evaluate the effect on teeth exposure.

The findings emphasize the importance of patient education regarding expected esthetic changes post‐procedure and the role of soft tissue dynamics in treatment planning. Moreover, understanding the mechanisms behind tissue response and its effect on dental exposure may provide valuable insights for improving treatment strategies and achieving more predictable results.

This study has several limitations. The small sample size and restriction to young female participants limit generalizability to other age groups, males, and ethnic populations. The absence of a control group makes it difficult to distinguish treatment effects from natural variability in posed smiling over time, and the lack of intra‐ and inter‐rater reliability.

Although 3D facial scanning allows objective soft‐tissue assessment, minor measurement errors and inter‐session variability cannot be excluded, particularly given the sensitivity of tooth exposure to subtle changes in lip posture and muscle activity. Smiles were posed rather than spontaneous, and reliability testing was not performed.

Finally, the 4‐week follow‐up reflects only early post‐treatment changes and does not capture the longer‐term behavior of HA fillers. Larger, controlled studies with longer follow‐up are needed to confirm the clinical relevance of these findings.

## 5. Conclusions

Within the limitations of this observational study, HA lip augmentation was associated with small, statistically significant changes in anterior tooth exposure during smiling. The observed reductions in central incisor and canine display were of modest magnitude and should be interpreted with caution, particularly in the absence of a control group and given the short follow‐up period. These findings suggest that changes in lip volume may subtly influence smile‐related dental parameters rather than producing pronounced clinical effects. Larger, controlled, and longer‐term studies are needed to confirm these observations and to better define their clinical relevance in esthetic dental planning.

## Funding

The authors received no financial support for the research, authorship, and/or publication of this article.

## Conflicts of Interest

The authors declare no conflicts of interest.

## Data Availability

The data that support the findings of this study are available upon request from the corresponding author. The data are not publicly available due to privacy or ethical restrictions.
